# Activation of PDE10 and PDE11

**DOI:** 10.1186/1471-2210-11-S1-P36

**Published:** 2011-08-01

**Authors:** Ronald Jäger, Corina Russwurm, Doris Koesling, Michael Russwurm

**Affiliations:** 1 Institut für Pharmakologie und Toxikologie, Medizinische Fakultät, Ruhr-Universität Bochum, 44780 Bochum, Germany

## 

Phosphodiesterases (PDEs) are critically involved in the determination of intracellular cyclic nucleotide levels. PDEs are heterodimers containing a conserved C-terminal catalytic domain and an N-terminal regulatory domain. Within their regulatory domain, 5 PDE families contain GAF domains, which are potential cyclic nucleotide binding domains. cGMP is known to activate PDEs 2 and 5.

Also PDE10 and 11 contain GAF domains in their N termini. However, the functional role of these domains remains a matter of debate. In chimeric constructs of PDE GAF domains and cyanobacterial adenylyl cyclase catalytic domains, cAMP and cGMP activated constructs containing the PDE10 and PDE11 GAF domains, respectively. On the other hand, binding of cAMP and cGMP was claimed not to activate the PDE10 and PDE11 holoenzymes.

Here we used synthetic ligands identified by fluorescence resonance energy transfer (FRET)-based analysis of isolated GAF domains to demonstrate that PDE10 and 11 holoenzymes are activated by their GAF domains. Furthermore, we show that PDE10 is activated by cAMP and that PDE11 albeit sensitive to synthetic GAF ligands is not activated by the physiological nucleotides cGMP and cAMP.

